# Domain similarity based orthology detection

**DOI:** 10.1186/s12859-015-0570-8

**Published:** 2015-05-13

**Authors:** Tristan Bitard-Feildel, Carsten Kemena, Jenny M Greenwood, Erich Bornberg-Bauer

**Affiliations:** Institute for Evolution and Biodiversity, University of Münster, Hüfferstr. 1, Münster, Germany

**Keywords:** Domain, Domain similarity, Orthology, Similarity

## Abstract

**Background:**

Orthologous protein detection software mostly uses pairwise comparisons of amino-acid sequences to assert whether two proteins are orthologous or not. Accordingly, when the number of sequences for comparison increases, the number of comparisons to compute grows in a quadratic order. A current challenge of bioinformatic research, especially when taking into account the increasing number of sequenced organisms available, is to make this ever-growing number of comparisons computationally feasible in a reasonable amount of time. We propose to speed up the detection of orthologous proteins by using strings of domains to characterize the proteins.

**Results:**

We present two new protein similarity measures, a cosine and a maximal weight matching score based on domain content similarity, and new software, named porthoDom. The qualities of the cosine and the maximal weight matching similarity measures are compared against curated datasets. The measures show that domain content similarities are able to correctly group proteins into their families. Accordingly, the cosine similarity measure is used inside porthoDom, the wrapper developed for proteinortho. porthoDom makes use of domain content similarity measures to group proteins together before searching for orthologs. By using domains instead of amino acid sequences, the reduction of the search space decreases the computational complexity of an all-against-all sequence comparison.

**Conclusion:**

We demonstrate that representing and comparing proteins as strings of discrete domains, i.e. as a concatenation of their unique identifiers, allows a drastic simplification of search space. porthoDom has the advantage of speeding up orthology detection while maintaining a degree of accuracy similar to proteinortho. The implementation of porthoDom is released using python and C++ languages and is available under the GNU GPL licence 3 at http://www.bornberglab.org/pages/porthoda.

**Electronic supplementary material:**

The online version of this article (doi:10.1186/s12859-015-0570-8) contains supplementary material, which is available to authorized users.

## Background

Bioinformatic programs to detect orthologous proteins have become indispensable in everyday biological research. These programs allow the classification of protein sequences sharing evolutionary origins and provide a better understanding of the evolutionary forces acting on organisms.

Many algorithms have been developed to predict clusters of orthologous proteins. The algorithms can be grouped into two families: tools using phylogeny-based methods [1,2] and tools using pairwise sequence comparison and clustering approaches [3-5]. The core component of these methodologies relies on a time-consuming pairwise comparison of sequences and, accordingly, scales quadratically with the number of sequences being tested for orthology. This step can soon become non-permissive since the number of organisms sequenced is rapidly increasing.

A solution to reduce the large computational time burden is to decrease the number of pairwise comparisons. Instead of comparing all proteins against all, proteins could be clustered together into smaller groups. These smaller groups correspond to a sub-search space in comparison with the huge orthology search space created by the all-against-all pairwise comparison. However, the creation of the clusters of proteins should be fast and therefore should not rely on amino acid sequence comparisons. A coarse-grained, biologically relevant description of the protein should instead be used, for example, one based on protein domains. Domains correspond to conserved portions of sequences that can be found in different proteins and in combination with identical or other domains. A domain can be seen as an extension of François Jacobs’ statement that nature tends to reuse already existing material to create novelties [6]. By their arrangement in a specific combination, domains provide a way for organisms to create new functions from already existing material. A domain arrangement is defined as the combination of domains in a protein and so is composed of at least one domain. Protein domain modularity makes domains the unit of proteome evolution [7]. Moreover, since the number of new domain arrangements increases faster than the number of new domains detected [8], molecular diversity, similarity and divergence can be described by domain arrangements (DAs).

Indeed, DA similarity analyses have been successfully used to study the evolutionary relationship between proteins [7,9,10] from a phylogenetic point of view and have been used to predict orthologous proteins using phylogenetic information [11]. Furthermore, the comparison of DAs, as linear (RADS [12]) and circular permutations (RASPODOM [13]), have been proven to enhance the performance of classical bioinformatic methodologies.

Methods to compare and group proteins based on the similarity of their DAs have already been proposed [14-17]. All these methods use a binary representation for the comparison of two domains, i.e. two domains are recorded as either being identical or different. Such a representation lacks flexibility when comparing DAs with evolutionary divergence in one or several of their domains. For example a [PBC;Homeobox] DA, with a PBC domain at the N terminus and a Homeobox domain at the C terminus, will be considered different from a [PBC;Homeobox_KN]-DA, even though Homeobox and Homeobox_KN are two similar homeobox transcription factor domains. Moreover, a comparison between domains and amino acid sequence-based methods to correctly group proteins into families has shown that a sequence-based method introduced by Song et al. [18,19] outperforms domain-based methods [18]. Improvement in the accuracy of domain-based methods are therefore needed before using domain-based similarity measures for orthology prediction.

Here, a new method is proposed using the evolutionary information carried by the domain content of proteins and using a domain-domain similarity score in a continuous space. The continuous similarity scores allow non-identical domains to be similar, thus removing the problem of binary similarity measures. Two scores are introduced to compute the similarity between DAs using this continuous similarity measure between domains.

First, the scores are evaluated, independently of the orthology detection method, against manually created and curated benchmarks: a dataset used by Song et al. [18] and the OrthoBench protein family dataset [20]. The goal of this evaluation is to compare the sequence-based score, which previously outperformed DA-based binary similarity scores, with the similarity measures developed here.

Next, the best DA similarity measure is determined, then used in combination with an orthology detection method and evaluated on a test case of 32 arthropod proteomes.

## Methods

The methods comprise two parts. The first part describes the development of the domain-based similarity measures between proteins. Different measures are tested on reference classifications of protein families and machine learning techniques are used for the comparison and the evaluation of the measures. The second part describes the implementation of porthoDom, the wrapper around proteinortho used to produce clusters of ortholgous proteins. The porthoDom and proteinortho methods are then evaluated on a real dataset of proteomes and compared against an external database of orthologous proteins as a blind validation.

### Creation and evaluation of the similarity measures

All the measures developed rely on a pairwise domain similarity matrix. The similarity matrix between domains is created based on the direct comparison of domain models and provides a continuous similarity score.

The different similarity measures are then tested on three manually created and curated benchmarks and the results compared between each measure and the NeighbourhoodCorrelation (NC) method [18,19], which has previously been shown to outperform binary domain-based scores.

The methods are compared using ROC curves and AUC scores and subsequently, the best-performing method is selected to be used as a pre-processing step of the protein orthology detection method.

#### Domain similarity matrix

To improve the comparisons between domains, a domain-domain similarity matrix was developed. The matrix was created using domains in Pfam-A [21] version 27.0 (containing 14831 Hidden Markov Models, or HMMs) but the method can be used with any other HMM database. The similarity between domains is computed using the HHsearch tool [22]. Each HMM corresponding to a domain in the Pfam-A database is aligned against all the models of the Pfam-A database, resulting in 14831^2^ pairs of aligned HMMs. Furthermore, the probability of a true positive match is used as a similarity score, as recommended in [22]. The true positive match value corresponds to the probability that two compared models are homologous or that the sequences share a good structural alignment. The scores returned by HHsearch range between [0,100], but 95% of the scores are equal to or below 1. To reduce the size of the similarity matrix, only scores between [1,100] are stored (see Additional file 1).

The matrix is available on the web site alongside the software.

#### Cosine similarity (COS) measure

A cosine similarity measure is implemented to compute the distance between two DAs of any length. The cosine measure is a similarity measure often used for high dimensional spaces. Therefore, the measure is useful in the comparison of domain contents, as the number of domains contained in the Pfam database is higher than our classical three-dimensional space.

The cosine measure is computed between two vectors *x* and *y* as follows:

(1) $$\begin{array}{@{}rcl@{}} cos = \frac{x \cdot y}{ \parallel x\parallel \parallel y \parallel} \end{array} $$

The cosine similarity between two proteins is calculated as follows: Let X and Y be two proteins of respective domain arrangements: *ABCC* and *DBBCE*, then *D*_*X*_ and *D*_*Y*_ correspond to two sets, each one made up of the unique domains extracted from their respective proteins. The domain universe set is defined as the union of the two sets, $U_{\textit {XY}} = D_{X} \bigcup D_{Y}$. For example, let protein X correspond to the domain set *D*_*X*_={*A*;*B*;*C*} and protein Y to *D*_*Y*_={*B*;*C*;*D*;*E*}. The domain universe set then becomes *U*_*XY*_={*A*;*B*;*C*;*D*;*E*}.

For each protein, a similarity vector of the size of the domain universe set is created. In this example, two vectors of length 5 will be created for protein X and protein Y respectively, noted $\overrightarrow {X_{U}}$ and $\overrightarrow {Y_{U}}$. The scores at the different positions *i* are defined as follows:

(2) $$\begin{array}{*{20}l} \overrightarrow{X_{Ui}} = \left\{\begin{array}{ll} 1, & \text{if}\ U_{XYi} \in D_{X} \\ S(X_{Ui}, D_{X}) & \text{otherwise} \end{array} \right. \end{array} $$

(3) $$\begin{array}{*{20}l} S(X_{Ui}, D_{X}) = max(X_{Ui}, D_{Xi}),~\forall D_{Xi} \in D_{X} \end{array} $$

If a domain is present in a protein, the similarity is equal to 1, otherwise the similarity will be taken as the similarity value between this domain and the most similar domain in the corresponding protein.

In the example, let domains A and D be evolutionarily closely-related domains, (*S*(*A*,*D*)=0.8), and domain E, a domain sharing no similarity with any of the domains of the protein X, (*S*(*A*,*E*)=0, *S*(*B*,*E*)=0, *S*(*C*,*E*)=0). The universe vector of protein X will then be $\overrightarrow {X_{U}}=\{1.0, 1.0, 1.0, 0.8, 0.0\}$, where A, B, and C are present in *D*_*X*_, D and A are similar and E shares no similarity with the other domains. The universe vector of protein Y $\overrightarrow {Y_{U}}=\{0.8, 1.0, 1.0, 1.0, 1.0\}$, where A and D are similar and B, C, D, E are present in *D*_*Y*_ These two vectors will lead to closely related positions in domain space. In consequence, the cosine similarity between two universe vectors of two proteins with no domain in common will be 0, and the cosine similarity between two universe vectors of two proteins with the same domains but different arrangements will be 1.

#### Maximal weight matching (MWM) measure

MWM is a classic measure of pairwise vertex assignment in graph methodology and is based on edge similarity. The algorithm is used in graph theory to solve optimisation problems of pairing. Here, the implementation from the networkx python library is used; for details see [23,24].

The MWM algorithm is used as a method to optimise the similarity between domains of two different proteins. The domains of two proteins correspond to the vertices of the graph. Edges are created between all of the domains of the protein X and all of the domains of the protein Y. No edges are allowed between domains of the same protein. A weight is put on each edge corresponding to the similarity computed between the two domains linked by the edge. The weights correspond to the scores from the similarity matrix. This results in the creation of a bipartite graph. The MWM algorithm then selects the set of edges with the maximal total weight, where each node can only be chosen once. Continuing with the example DAs, *D*_*X*_ and *D*_*Y*_, the set of vertices *V* and the set of edges *E* correspond to:

(4) $$\begin{array}{*{20}l} V=\{A_{X}; B_{X}; C_{X}; B_{Y}; C_{Y}; D_{Y}; E_{Y}\} \end{array} $$

(5) $$\begin{array}{*{20}l} E=\{ (A_{X}, B_{Y}); (A_{X}, C_{Y});... ; (C_{X}, D_{Y}); (C_{X}, E_{Y})\} \end{array} $$

A domain of protein X can only be grouped with one domain of protein Y, so that the set of edges selected by the MWM algorithm will be:

(6) $$\begin{array}{@{}rcl@{}} E'=\{(A_{X}, D_{Y}); (B_{X}, B_{Y}); (C_{X}, C_{Y})\} \end{array} $$

The final similarity score is then computed as the sum of the edge weights normalized by the size of the longest protein set.

#### Adding domain order to the COS and MWM measures

Considering that the order of domains in a protein is important and contains a strong phylogenetic signal which indicates protein functional similarity [9,25,26], domain order information is added to the protein similarity measures.

When only the domain content is compared between two proteins, the measure will here be referred to as an order 1 measure (later on abbreviated as *O*1). The COS and MWM measures described above correspond to such *O*1 measures (*C* *O* *S*_*O*1_ and *M* *W* *M*_*O*1_). Similarity measures with an order of 2 (*O*2) are introduced by using pairs of consecutive domains instead of single domains.

Using the cosine computation example, the set of pairs considered for protein X is *D*_*X*_={*A* *B*;*B* *C*;*C* *C*} and the set of pairs for protein Y is *D*_*Y*_={*D* *B*;*B* *B*;*B* *C*;*C* *E*}. The domain universe set is then:

(7) $$\begin{array}{@{}rcl@{}} U_{XY} = \{AB; BC; CC; DB; BB; CE \} \end{array} $$

The scores at the different positions of the $\overrightarrow {X_{U}}$ and $\overrightarrow {Y_{U}}$ universe vectors of the two proteins are computed using the mean similarity between pairs instead of the direct domain-domain similarity measure:

(8) $$\begin{array}{@{}rcl@{}} S_{p}(AB, DB) &= 0.5 \times (S(A,D) + S(B,B)) \end{array} $$

The mean similarities replace the values in the cosine vector (equation 2) and the weights on the edges for the MWM method (*C* *O* *S*_*O*2_ and *M* *W* *M*_*O*2_ respectively).

#### Adding a weight to the COS and MWM measures

Next, the effect of a weighting scheme is evaluated for the COS and MWM measures. The purpose of applying the weighting scheme is to enhance the weight of highly similar domains in the computation of the final similarity measure. The scores produced with an *O*2 parameter tend to be higher than the scores computed with an *O*1 parameter. This effect is due to a smaller number of comparisons in the *O*2 domain universe set and to the usage of a mean similarity when pairs of domains are compared.

If a domain pair *AB* is compared to the pair *AC*, and *C* and *B* do not share any similarity, the similarity between the two pairs will be 0.5 according to equation 8. A mean similarity of 0.5 with an *O*1 parameter corresponds to a direct medium similarity score between domains. Therefore, the scores computed with an *O*2 parameter can result in the grouping of proteins of two different clusters by creating false links between them. To give more importance to scores computed between similar pairs with an *O*2 parameter, the scores are weighted depending on the order parameter used. More details are given in the Additional file 1 regarding the weighting scheme implementation and its effect on the scores (Additional file 1: Figure S2).

In total, eight different similarity measures are tested: *C* *O* *S*_*O*1_, *C* *O* *S*_*O*2_, *M* *W* *M*_*O*1_ and *M* *W* *M*_*O*2_ with and without a weighting scheme applied.

### The protein domain content similarity measure as a pre-processing step for orthology

After selecting the best-performing similarity measure, the measure is implemented in a piece of software built around proteinortho. In this section, an explanation of the software implementation and testing is provided.

#### porthoDom

The newly-developed method, named porthoDom, is made up of a python wrapper and a C++ program for proteinortho [4] (version 4.26). The aim of porthoDom is to use the domain content similarity between proteins to reduce the initial search space by clustering proteins with similar domains together. Clusters of proteins with similar domains form “search sub-spaces”. Orthology detection is applied to the group of sequences belonging to the same sub-space.

The python implementation uses the numpy (version 1.8.1), and networkx (version 1.8.1) libraries. The clustering is done using the kmedoids algorithm of Pycluster (version 1.52) [27].

In detail, the method follows these steps:

- Starting with a list of proteomes, proteins are annotated by the pfam_scan.pl script and the DAs of the proteins are extracted. Alternatively, the user can provide precomputed annotation files of the proteomes.
- From the list of DAs of all the proteins in all proteomes, a list of the unique DAs is created. The pairwise similarity between all the unique DAs is then computed.
- Unique DAs are clustered using the kmedoids algorithm, set to look for 100 clusters by default. The clusters correspond to sub-spaces of the initial all-against-all protein search space.
- Protein and amino-acid sequences corresponding to each sub-space are retrieved and processed into new files and folders onto which proteinortho can be applied independently.
- Proteinortho is then used on each sub-space, at the sequence level.
- A classical proteinortho formatted output file is created by gathering the results of all runs of proteinortho.

*C* *O* *S*_01_ is the default domain content similarity measure implemented in porthoDom, but other measures can be set optionally as a parameter. Default parameters of proteinortho can be changed by providing a configuration file. The wrapper can also start the orthology prediction on a precomputed dataset with different parameters, a useful and conserved original feature of proteinortho.

#### Collapsing of domain repeats

In biological datasets, a possible bias in domain content similarity computation can result from tandem repetition of domains [28-30]. To evaluate the efficiency of porthoDom for grouping DAs with repeats, the similarity between DAs can be computed both with tandem repeats collapsed and with the original DA. A protein with a DA {*A*;*B*;*B*;*C*;*B*;*C*} becomes {*A*;*B*;*C*;*B*;*C*} where tandem repeats are collapsed.

## Results and discussion

### Evaluation of the COS and MWM similarity measures

In the following section, the developed similarity measures, COS and MWM, are evaluated against the NeighbourhoodCorrelation (NC) method [18,19]. The NC methodology is based on sequence comparison and has been shown to provide better protein family classification results when compared to a domain-based similarity measure [18].

Two manually curated datasets are used for evaluation: a dataset used by Song et al. [18] (later on abbreviated to SD) and the OrthoBench dataset [20] (OB). The SD dataset comprises 20 protein families whereas the OB dataset comprises 69 families. More details on the dataset can be found in [18,20]. All proteins without DAs are removed from the SD and OB datasets, respectively 5 out of 1816 and 57 out of 1695, for easier interpretation of the comparison between the NC, COS and MWM measures. Moreover, one of the families in the SD, the kinase family, is much larger than the other families and due to its size, can create a classification bias [18,19].

In accordance with previous studies [18,19], the SD dataset is analysed both with the kinase family (SD ^+^) and without (SD ^−^). The measures are then evaluated on both datasets and any potential bias induced by the presence of the kinase family in the SD ^+^ can be detected by comparison with the results obtained from SD ^−^.

The COS, MWM and NC measures are evaluated on the SD ^+^, the SD ^−^ and the OB datasets using receiver operating characteristic (ROC) curves and area under the curve (AUC) scores. The ROC curves and AUC scores are used to compare the true and false positive rates (TPR and FPR) of the COS, MWM and NC similarity measures. These comparisons are made using the standard python machine learning library scikit-learn [31].

**Figure 1:**
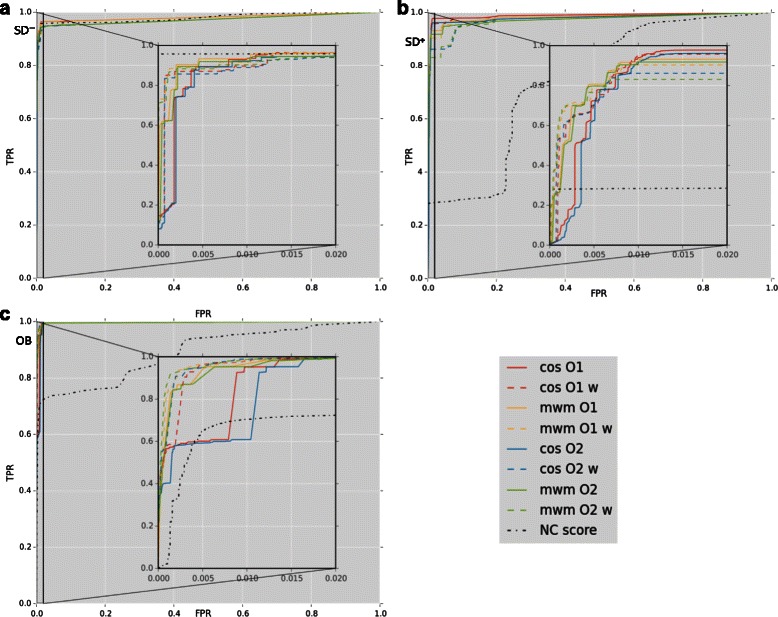
ROC curves. ROC curves of the developed COS and MWM measures, and of the NC method against the SD ^−^ dataset (panel **a**), the SD ^+^ dataset (panel **b**) and the OB dataset (panel **c**). For each panel, the left plots correspond to the full ROC curves and the right plots to a zoomed in subsection along the *x* axis. *C* *O* *S*_*O*1_, *C* *O* *S*_*O*2_, *M* *W* *M*_*O*1_ and *M* *W* *M*_*O*2_ are evaluated with weighting (*w*) or without. The influence of the kinase family in the SD ^+^ dataset on the sequence similarity based method (NC) is clearly seen in panel **b**.

Figure 1 displays the ROC curves for the COS and MWM measures, with or without weighting, and the NC score. When evaluating the COS, MWM and NC measures against the SD ^−^ (Figure 1a), the three measures give similar results. The *O*1 and *O*2 parameters with or without weighting also produce comparable results. However, for equivalent FPR, the TPR of the ROC curves is slightly lower for all similarity measures (Figure 1b) for the SD ^+^. The TPR over FPR reduction is far more pronounced for the NC measure than for the COS and MWM measures. The reduction in TPR over FPR for the SD ^+^ compared to the SD ^−^ for all of the measures can be explained by the presence of the kinase family in SD ^+^ as the overall TPRs and FPRs are strongly influenced by the TPR and FPR of the kinase family. Similarly to the SD ^−^ dataset, the *O*1 and *O*2 parameters with or without weighting do not produce different results for the SD ^+^ dataset.

The ROC curves of the COS, MWM and NC measures against the OB dataset (Figure 1c) are similar to the curves of the SD ^+^ dataset. In the OB dataset, the TPR over FPR curves of the NC measure are far lower than the other measures. A slightly higher TPR over FPR can be detected in this dataset for the COS and MWM measures with a weight parameter, with a stronger TPR over FPR difference between the COS measures with and without weight.

**Figure 2:**
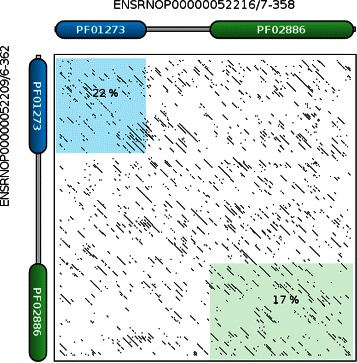
Dotplot with domain visualisation of two proteins belonging to the PLUNC family (ENSRNOP00000052209 and ENSRNOP00000052216). The shadowed areas correspond to the sequence identity between the two sequences. Although they share the exact same DA, their sequence similarity is very low (20.8%). Run with the needle program of the EMBOSS package [35]. Dotplot was produced with the DoMosaics software [36].

An example in which domain-based similarity scores are more efficient at grouping proteins of the same family than sequence-based similarity scores is presented in Figure 2. The sequences of two proteins, ENSRNOP00000052209 and ENSRNOP00000052216, belonging to the PLUNC family from the OB dataset are compared. PLUNC proteins (a member of the bactericidal permeability-increasing (BPI)-like proteins) are involved in defence against bacteria and are well-known for their fast evolution and low sequence similarity [32]. The sequence-based NC measure has difficulty correctly retrieving the members of the PLUNC family due to their low sequence similarity. However, the proteins of the PLUNC family are only composed of two domains (PF01273, PF02886). The proteins of the family can be made of one or both of these domains. The PLUNC proteins are clearly classified as member of the same family by the COS and MWM methods with high similarity scores.

A comparison of the different measures is also performed based on AUC scores. The AUC scores are computed from the TPR and FPR of the different measures. Table 1 summarizes the performances of the COS, MWM and NC measures on the three different datasets (SD ^+^, SD ^−^, OB).

**Table 1:** **AUC scores for all methods against the SD** ^**−**^ **, the SD** ^**+**^ **and the OB datasets**

**Method**	**AUC (SD** ^**−**^ **)**	**AUC (SD** ^**+**^ **)**	**AUC (OB)**
NC	0.993	0.844	0.919
*C* *O* *S*_*O*1_	0.979	0.987	0.994
*C* *O* *S*_*O*2_	0.971	0.978	0.992
*C* *O* *S*_*O*1_ w	0.98	0.987	0.996
*C* *O* *S*_*O*2_ w	0.971	0.973	0.996
*M* *W* *M*_*O*1_	0.98	0.982	0.996
*M* *W* *M*_*O*2_	0.972	0.974	0.996
*M* *W* *M*_*O*1_ w	0.98	0.981	0.996
*M* *W* *M*_*O*2_ w	0.972	0.969	0.997

The AUC scores are computed from the TPR and FPR of the different measures. The scores reflect the quality of the COS, MWM and NC measures for protein family classification. An AUC score of 1 corresponds to a perfect classification of the dataset. All methods produce a very good AUC score, a small general advantage can be observed for the methods using an order 1 parameter. Cosine methods have better performances on the SD ^+^ dataset and the MWM methods perform generally better on the SD ^−^ dataset and on the OB dataset. Using the weighted version of the COS or MWM measure only improves the performance on the OB dataset.

The COS and MWM measures outperform the NC method for SD ^+^ and OB in terms of AUC scores. The AUC scores from SD ^−^ are similar for all measures tested, but are slightly better for NC. The AUC score of the NC measure from SD ^+^ is lower than the AUC scores of the COS and MWM measure and confirm that the NC classification is more sensitive to the presence of the kinase family in SD ^+^.

AUC scores are similar between the different COS and MWM measures. When COS and MWM are compared with the same order and weighting scheme, the MWM measures perform better on the SD ^−^ dataset than the COS measures. However, the opposite can be seen for the SD ^+^ dataset where the COS measures have higher AUC scores than the MWM measures.

On the OB dataset, MWM and COS measures produce very similar AUC scores, slightly higher in general for the MWM measures.

The *C* *O* *S*_*O*1_ and *M* *W* *M*_*O*1_ measures perform as well or better than the *C* *O* *S*_*O*2_ and *M* *W* *M*_*O*2_ measures on all datasets. Moreover, the weighting scheme slightly increases the performance of the COS and MWM measures on the OB dataset.

As the COS and MWM produce highly similar results, in order to select one domain content similarity measure, the complexity of both measures are compared. MWM has a complexity of *O*(*V* ^2^ *E*), with *V* being the number of vertices and *E* the number of edges, whilst the COS measure has a linear complexity. Therefore, the computational complexity of the COS measure is lower than that of MWM. Based on these results, the COS algorithm is chosen for the preprocessing stage of proteinortho.

### Evaluation of protein orthology detection

Next, a direct application of the domain content similarity measure to protein orthology detection is presented with an evaluation of the predicted orthologous groups of proteins. Proteomes of 32 arthropods were downloaded from the Ensembl Metazoan website, version 20 (Additional file 1: Table S1). The arthropod proteomes constitute a good test case due to the density of the clade and their annotation qualities.

PfamA-27.0 with the pfam_scan.pl annotation pipeline is used to assign domains to the protein sequences. Protein orthologous detections are performed on the arthropod dataset using the porthoDom and proteinortho software.

The quality evaluation of the orthology prediction is accomplished by comparisons of the protein orthology predictions with an external dataset used as a reference, OrthoDB (version 5). As the detection of orthologous proteins is not a trivial task, different methodologies will often lead to different results, so the comparisons of proteinortho and porthoDom to an external reference allow the methodologies to be evaluated strictly on their general behaviour.

Predicted groups of orthologous proteins produced by proteinortho and porthoDom (abbreviated as PGOP for predicted group of orthologous proteins), are compared to the groups of orthologous proteins in the OrthoDB dataset (abbreviated as RGOP for reference group of orthologous proteins).

A PGOP can be classified into five non-overlapping categories, depending on the relationship between the PGOP and the RGOP (see Additional file 1: Figure S2). The five categories are:

- a superset: a PGOP is a superset compared to an RGOP if all the proteins of an RGOP are present in the PGOP and some of proteins of the PGOP are not present in the RGOP.
- a subset: a PGOP is a subset compared to an RGOP if the proteins of the PGOP are all present in the RGOP but some other proteins of the RGOP are not in the PGOP.
- identical: a PGOP is identical to an RGOP.
- absent: a PGOP cluster is absent compared to the list of RGOP clusters if no proteins of the PGOP cluster are found in the RGOPs.
- new: a PGOP cluster is new compared to the list of RGOP clusters if the PGOP cluster is composed of parts of several RGOPs.

The influence of the following porthoDom parameters on these categories are evaluated: a domain content similarity cut-off (0.5, 0.7), domain content similarity order (*O*1, *O*2) and collapsing or not collapsing of domain repeats. Proteinortho is used with the default parameters in both porthoDom and the standalone version. The results of the proteinortho and porthoDom predictions are evaluated against the reference dataset and against each other.

**Figure 3:**
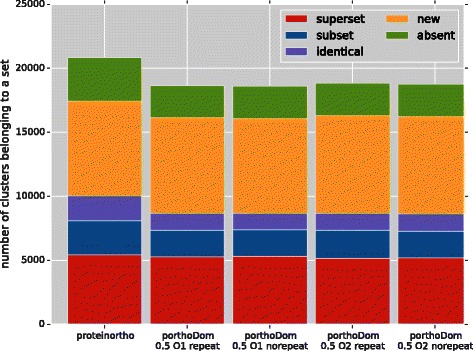
Results of comparisons between porthoDom or proteinortho against the OrthoDB database. Different parameters are used for the domain content similarity step of porthoDom and the default parameters of proteinortho are used for both methods. The parameters are: a domain content similarity cut-off of 0.5, a domain content similarity of O1 corresponding to single domain comparisons, or O2 corresponding to the comparison of pairs of domains, and an option collapsing or not of tandem domain repeats. The different parameters have little influence on porthoDom due to the robustness of the domain content similarity method.

The comparison of the Proteinortho and porthoDom results against the reference dataset for the domain content similarity cut-off of 0.5 are presented in Figure 3; results for the domain content similarity cut-off 0.7 can be found in Additional file 1: Figure S3. All combinations of porthoDom parameters clearly show similar trends in the proportion of the five different categories. This result is similar to the ROC and AUC analyses in which the change of parameters also had little influence on the results and always produced good classification scores. The robustness of the score is likely an effect of the pairwise domain scoring stored in the domain-domain similarity matrix.

The numbers of clusters in each category are provided in Table 2. The total numbers of protein clusters compared to the reference dataset are similar between proteinortho and porthoDom. The proportion of clusters that are subsets of the reference is equivalent between the two methodologies. The biggest difference comes from the number of identical and superset groups. Superset clusters tend to be more numerous for the porthoDom results than for proteinortho and the number of identical clusters is higher for proteinortho than for porthoDom. porthoDom creates a higher percentage of new clusters than proteinortho but also finds a lower number of absent clusters for all combinations of porthoDom parameters.

**Table 2:** **Number and percentage of clusters in the different evaluation groups for proteinortho and porthoDom**

**Method**	**Proteinortho**	**PorthoDom**	**PorthoDom**	**PorthoDom**	**PorthoDom**
		**(O1, norepeat)**	**(O1, repeat)**	**(O2, norepeat)**	**(O2, repeat)**
Superset (%)	5442 (26.13)	5311 (28.53)	5274 (28.26)	5172 (27.58)	5161 (27.4)
Subset (%)	2639 (12.67)	2054 (11.03)	2077 (11.13)	2104 (11.22)	2171 (11.52)
Identical (%)	1945 (9.34)	1311 (7.04)	1333 (7.14)	1351 (7.21)	1328 (7.05)
New (%)	7419 (35.62)	7396 (39.73)	7445 (39.89)	7580 (40.43)	7622 (40.45)
Absent (%)	3383 (16.24)	2545 (13.67)	2536 (13.59)	2543 (13.56)	2559 (13.58)

The domain content similarity cut-off of porthoDom was set to 0.5 and different combination of parameters affecting order (O1, O2) and repeats (with or without) were tested.

Varying porthoDom parameters only has a small influence on the results. The *C* *O* *S*_*O*2_ domain content similarity used in porthoDom is more stringent than the *C* *O* *S*_*O*1_ similarity. The small decrease in the proportion of identical, subset and superset clusters with the *C* *O* *S*_*O*2_ reflects the stringency of the choice of domain order parameter. All combinations of porthoDom parameters used are robust to the presence of repeats and repeat collapsing does not alter the proportions of clusters in each of the classification categories.

Identical, superset and subset clusters are the most important classes for the comparisons between PGOP and RGOP. These three classes reflect similar clusters between the prediction and the reference. Interestingly, porthoDom and proteinortho give a comparable total proportion of the three classes.

A direct comparison between the clusters computed by porthoDom and by proteinortho is provided in Additional file 1: Figure S5 (here PGOP refers to the porthoDom classification, and RGOP refers to the porteinortho classification). The comparison shows that the majority of clusters are identical between predictions. The influence of masking repeats can be seen in the number of subsets created. Masking domain repeats increases the number of clusters found by both porthoDom and proteinortho and lowers the number of subset clusters.

Finally, a different number of initial clusters specified for the k-medoids algorithm are tested: 10, 100, 500 and 1000, in combination with a domain content similarity cut-off of 0.5, a domain order parameter of *O*1 and no repeat collapsing. The number of initial clusters based on domain content can strongly influence the program run-time and the results of the prediction. If too few clusters are set, the gain in run-time will be negligible. However, if a high number of initial clusters are created, the search sub-spaces will be numerous, resulting in a decrease in computational time but potentially also in accuracy. Additional file 1: Figure S6 compares the time and the classification of clusters created by different initialization parameters against proteinortho.

The comparison shows that setting the initial number of k-medoids from 10 to 1000 decreases the run-time (4 times faster). However, the number of identical clusters between porthoDom and proteinortho is reduced from 13255 to 11102 (*k*=1000 and *k*=10 respectively, 2153 fewer cluster). The decrease in the number of identical clusters is accompanied by an increase in the number of superset, subset, new and absent clusters.

Currently, no automatic method is implemented to set the initial number of k-medoids but a reasonable number seems to be one twentieth of the number of unique domain arrangements present in the dataset.

Despite the differences coming from the direct comparison of the results, the comparisons against an external dataset give similar predictions. The orthology prediction power of porthoDom and proteinortho can therefore be considered as equivalent.

The porthoDom wrapper is designed to be similar to proteinortho. Once the pairwise sequence comparison is generated, it can be reused with different parameters without again calling the full pipeline. A time comparison is performed on a full pipeline run between porthoDom and proteinortho. It is first important to note however, that porthoDom requires an extra step compared to proteinortho: the domain assignment using the Pfam annotation script.

Table 3 summarizes the time needed for three different runs of proteinortho and porthoDom with and without pfam_scan.pl. The porthoDom wrapper, in its full version, is 2.5 times faster than proteinortho and 5.7 times faster without the pfam_scan.pl preprocessing. After domain assignment, the time needed to complete a porthoDom run is taken up by the proteinortho clustering on the sub-spaces of proteins with similar domain contents. Computing the pairwise similarity between all the DAs (approx. 24000) and clustering them takes around 20 minutes; the remaining time corresponds to data processing.

**Table 3:** **Running time in minutes of proteinortho and porthoDom with and porthoDom without pfam_scan.pl for the domain annotation**

**Name**	**Proteinortho**	**PorthoDom**	**PorthoDom**
		**(with pfam_scan.pl)**	**(no pfam_scan.pl)**
Run 1	1587	627	279
Run 2	1588	649	272
Run 3	1588	623	269
Mean	1587.6	633	273.3

The reduction of the computational time is a clear consequence of the replacement of the all-against-all protein comparisons by sub-space searching for orthologs. Even with the speed progress made on the recent HMMER3 package [33], the hmmscan software used by the pfam_scan.pl script for the domain detection step is a bottleneck in terms of computational time, requiring around 300 minutes to assign domains to a total of 32 arthropod proteomes on a 64 core computer. However, as domain annotation is now a very common analysis in comparative genomics, many proteomes already have precomputed annotations. Therefore, it is expected that in most cases the porthoDom package will provide a significant time advantage.

## Conclusion

Orthologous protein detection is a crucial bioinformatic methodology for a wide range of analyses. Most methods for protein orthology detection use a pairwise Blast [34] all-against-all comparison of the proteins belonging to two or more organism to detect orthologs. The amount of time needed for the all-against-all comparison is a classic bottleneck in the area of comparative genomics. To reduce time requirements, the protein search space can be reduced using a domain content similarity measure as a preprocessing step. Instead of computing a full pairwise all-against-all comparison, sub-groups of sequences are clustered according to their domain content similarity values, and pairwise comparisons are subsequently restricted to all proteins within a cluster against each other.

In this paper, two new such measures of protein similarity are presented based on a cosine distance and a maximal weight matching algorithm. The measures use domain contents of proteins and a new continuous similarity score between domains to compare proteins against each other. The accuracy of the two measures has been benchmarked on curated datasets and both show an ability to efficiently group proteins from the same family together.

The cosine measure, due to its better performance and its lower complexity, is chosen to be combined with the orthologous protein detection tool, proteinortho, in a method named porthoDom. It is also important to note that the developed methodology could in theory be combined with any other sequence-based orthologous proteins detection tool, such as OrthoMCL [3].

The crucial parameter of porthoDom appears to be the number of initial domain-based clusters creating the search sub-spaces. Too high a number of initial clusters will result in the creation of too many search sub-spaces, leading to fast predictions but very different results compared to a normal proteinortho analysis. As proteinortho orthology predictions are based on the E-value similarity between protein sequences from a Blast comparison, too many initial clusters will lead to too few cluster members and can impact the resulting E-values. In contrast, too few initial clusters will give orthology predictions similar to the predictions of proteinortho alone but without a significant increase in speed.

Direct comparison of the two methods shows that the majority of clusters created by porthoDom are identical to the clusters created by proteinortho. porthoDom also creates clusters that are subsets of proteinortho. This result seems to be the consequence of the proteins grouped by domain content; if a protein is not placed in the correct cluster during the domain-based step, the resulting clusters based on sequence similarity will be smaller. However, porthoDom and proteinortho results are similar when compared using an external reference database of orthologous proteins. These results emphasize that protein orthology detection is not a trivial task and that different software often produces different results. Therefore, the clustering results created by both methods should be considered equivalent.

porthoDom should be seen as a new class of method for orthology detection, using both domain and amino-acid sequence similarities to create groups of orthologous proteins. Moreover, the pre-filtering step based on domain content similarity speeds up orthology detection by a factor ranging from 2.5 to 5.7.

A drawback of the method is that not every protein may be assigned a domain and, consequently, many proteins may not be amenable to further processing. In the arthropod dataset, proteins with an annotated domain represent 65% of the total number of proteins. Nonetheless, the number of known domains is increasing and therefore reducing the number of proteins without annotation, as well as improving the annotation of existing proteins.

Our ever-increasing knowledge of domains should positively affect the precision and efficiency of domain-based orthology detection methods.

## Additional file

Additional file 1
**Supplementary information: Domain similarity based orthology detection.**
