# Supplementary material for: Domain similarity based orthology detection
Source: BMC Bioinformatics. 2015 May 13;16:154. doi: 10.1186/s12859-015-0570-8 (PMC4443542; doi:10.1186/s12859-015-0570-8)
Supplement: Additional file 1 — Supplementary information: Domain similarity based orthology detection. [file 12859_2015_570_MOESM1_ESM.pdf]

Supplementary information: Domain similarity based orthology detection

Construction of the similarity matrix

The similarity matrix is constructed from the results of HHsearch. A cut-off of 1 is set to remove values higher and equal to this threshold. Otherwise, the 14831 pairwise scores, $(n*(n-1))/2 = 109971865$, are too computationally expensive to keep. The remaining values of the sparse matrix are kept in a binary Compressed Row Storage (CRS) format.

Maximal weight matching algorithm

Figure S1 illustrates the maximal weight matching measure implemented to compare the domain content of two proteins. Panel A represents two proteins to compare of different domain content and domain length. Panel B illustrates the creation of the matching graph; each node corresponds to a domain and edges are set between the nodes of the red protein and the nodes of the blue protein. The weights on each edge correspond to the similarity between the red and the blue domains. The solid edges represent the selected edges by the maximal weight matching algorithm. Panel C corresponds to the computation of the similarity scores based on the edges selected by the maximal weight matching algorithm.

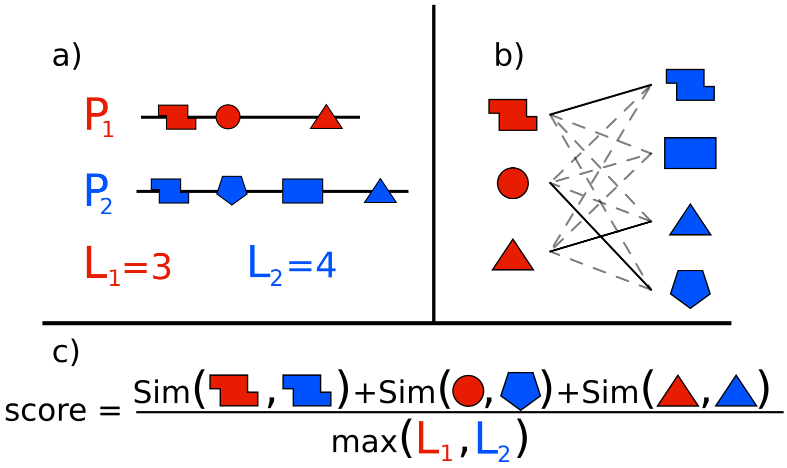

Figure S1: Maximal weight matching similarity measure between two proteins. a) Representation of two proteins with two different domain content; b) creation of the matching graph and selection of edges based on their weights; c) computation of the similarity measure.

Weighting scheme

Figure S2 displays the effect of weighting on the range of all possible values that the similarity measures can take (between 0 and 1). The weighting effect is less pronounced on higher similarity values, i.e. domains which are more similar. This increases the importance of similar domains

in the computation of the domain content similarity.

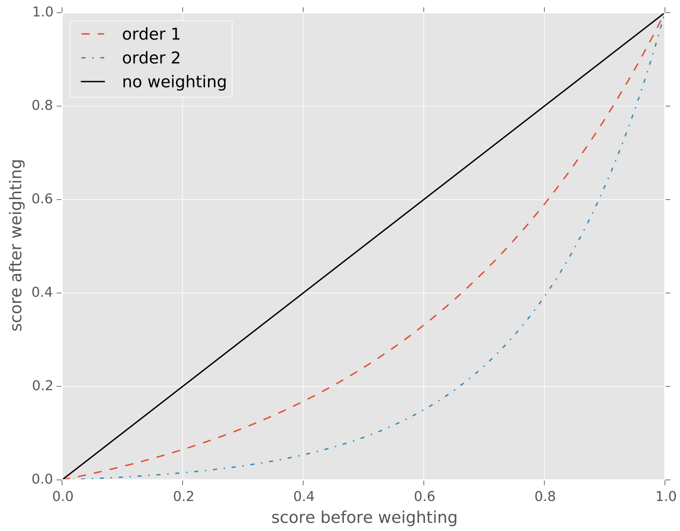

Figure S2: Effects of the weighting scheme on the original score for different similarity measures between domains or between pairs of domains. The red line (dashed line) corresponds to the transformation of scores with a domain order of 1. The blue line (dashed and dotted line) corresponds to the transformation of scores with a domain order of 2. The scores computed with pairs of domains are more affected by the weighting transformation.

The effect of the weighting scheme is more important for scores computed with a domain order of 2. Its purpose is to minimise the overall similarity when only one domain of the pair is similar to that in another pair. The weighting scheme is computed as follows:

$$a = 10^O \tag{1}$$

$$S' = \frac{(a^S - 1.0)}{a - 1.0} \tag{2}$$

where S' is the weighted similarity score and the label O is the order used for the computation of the original similarity measure S. Figure S2 shows the effect of the weighting scheme on the range of all possible values that the similarity measures can take. Note that with an $O2$ measure, if the two sets of domain pairs compared are too dissimilar, the score decreases faster than with an $O1$ similarity measure.

List of proteomes

Proteomes used have been downloaded from EnsemblMetazoan version 20.

Table S1: List of arthropod species used to compare proteinortho and porthoDom, with corresponding identification name and version.

Organism	identification name and version
Acromyrmex echinatior	aech_OGSv3.8
Acyrthosiphon pisum	GCA_000142985.2.20
Aedes aegypti	AaegL1.20
Anopheles gambiae	AgamP3.20
Apis mellifera	Amel4.0.20
Atta cephalotes	Attacep1.0.20
Bombyx mori	Bmor1.20
Camponotus floridanus	cflo_OGSv3.3
Culex quinquefasciatus	CpipJ1.20
Danaus plexippus	DanPle_1.0.20
Daphnia pulex	Dappu1.20
Drosophila erecta	dere_caf1.20
Drosophila grimshawi	dgri_caf1.20
Drosophila persimilis	dper_caf1.20
Drosophila melanogaster	BDGP5.20
Drosophila mojavensis	dmoj_af1.20
Drosophila pseudoobscura	HGSC2.20
Drosophila sechellia	dsec_caf1.20
Drosophila simulans	WUGSC1.20
Drosophila virilis	dvir_caf1.20
Drosophila yakuba	dyak_r1.3_FB2008_07.20
Drosophila willistoni	dwil_caf1.20
Harpegnathos saltator	hsal_OGSv3.3
Heliconius melpomene	Hmel1.20
Ixodes scapularis	IscaW1.20
Linepithema humile	lhum_OGSv1.2
Nasonia vitripennis	Nvit1.20
Pediculus humanus	PhumU1.20
Pogonomyrmex barbatus	pbar_OGSv1.2
Solenopsis invicta	sinv_OGSv2.2.3
Tribolium castaneum	Tcas3.20
Zootermopsis nevadensis	Znev.OGS.v2.2

Comparisons used to evaluate predicted groups of orthologous proteins and reference groups of orthologous proteins from the OrthoDB database.

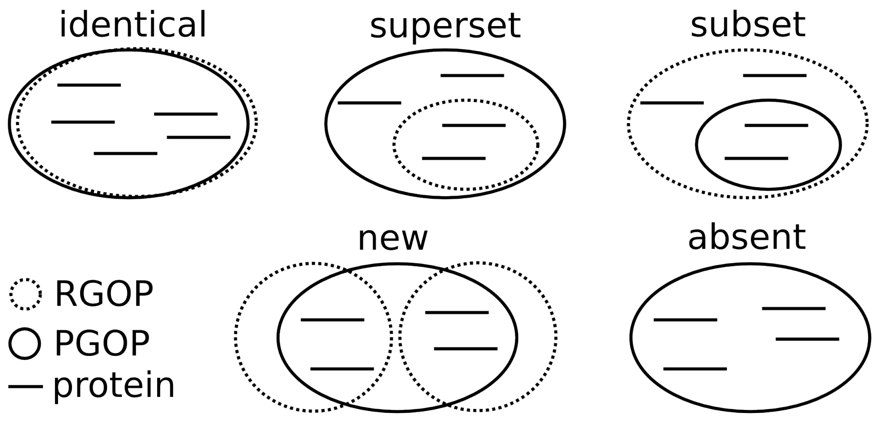

Figure S3: Definition of identical, superset, subset, new and absent categories when comparing a reference group of orthologous proteins (RGOP) from OrthoDB (dashed circles) with predicted group of orthologous proteins (PGOP) using proteinortho or porthoDom (solid circles). An identical cluster is found when the two sets of proteins, RGOP and PGOP, are equal. A superset is found when the PGOP is a superset of an RGOP. A subset is found when the PGOP is a subset of an RGOP. A PGOP cluster is absent from OrthoDB when none of its proteins are in an RGOP. Otherwise a new cluster is defined.

Protein orthology detection comparison

Higher similarity cut-off

Table S2 and Figure S4 show the results of the comparison between proteinortho and porthoDom with a domain content similarity cut-off of 0.7 and various parameters. The results of proteinortho are similar to the results of porthoDom with a similarity cut-off of 0.5. The influence of the cut-off is limited and proteinortho and porthoDom behave similarly against the external reference database OrthoDB.

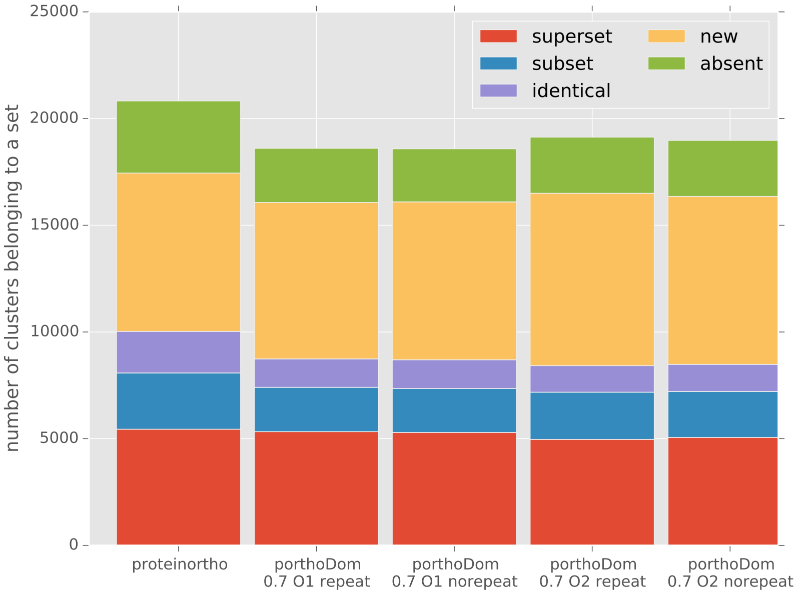

Figure S4: Results of comparisons between porthoDom and proteinortho against the OrthoDB database. Different parameters are used for the domain similarity step of porthoDom and the default parameters of proteinortho are used for both methods. The parameters are: a domain similarity cut-off of 0.7; a domain order of 1 (single domain comparisons); a domain order of 2 (domain pair comparisons); collapsing or not of tandem domain repeats.

Table S2: Number and percentage of clusters in the different evaluation group for proteinortho and porthoDom with a cut-off similarity of 0.7 and various parameters.

method	proteinortho	porthoDom (O1, norepeat)	porthoDom (O1, repeat)	porthoDom (O2, norepeat)	porthoDom (O2, repeat)
superset (%)	5442 (26.13)	5291 (28.47)	5329 (28.64)	5059 (26.66)	4967 (25.97)
subset (%)	2639 (12.67)	2060 (11.08)	2077 (11.16)	2154 (11.35)	2210 (11.55)
identical (%)	1945 (9.34)	1348 (7.25)	1334 (7.17)	1268 (6.39)	1245 (6.5)
new (%)	7419 (35.62)	7398 (39.81)	7329 (39.39)	7874 (41.49)	7622 (42.23)
absent (%)	3383 (16.24)	2488 (13.38)	2537 (13.65)	2621 (13.81)	2630 (13.75)

porthoDom versus proteinortho

Figure S5 presents a direct comparison of porthoDom and proteinortho. The comparison shows that most of the clusters are identical between predictions. The effect of collapsing tandem domain repeats can be seen in the number of subsets created. Collapsing domain repeats increases the number of clusters classified as identical between porthoDom and proteinortho and lowers the number of subset clusters.

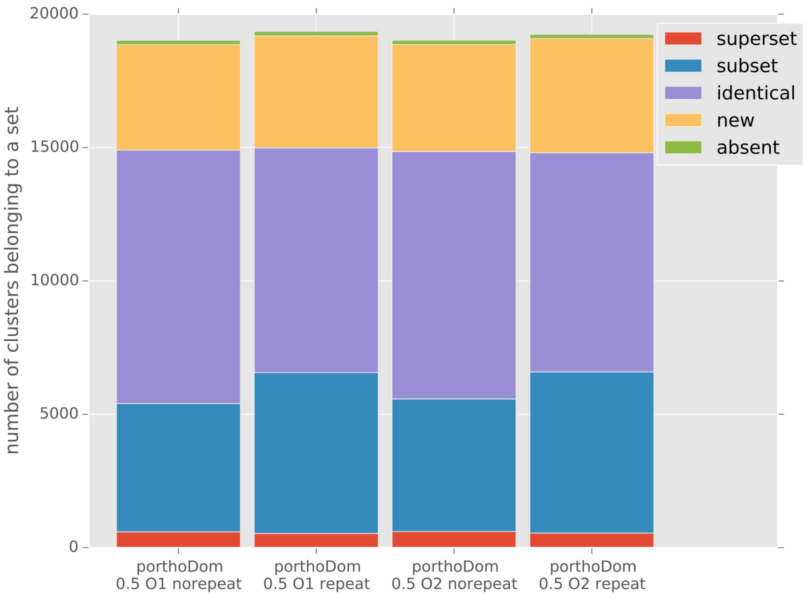

Figure S5: Results of comparisons between porthoDom against proteinortho. Different parameters are used for the domain similarity step of porthoDom and the default parameters of proteinortho are used for both methods. The parameters are: a domain similarity cut-off of 0.7; a domain order of 1 (single domain comparisons); a domain order of 2 (domain pair comparisons); collapsing or not of tandem domain repeats.

Effect of different initial numbers of k-medoids

Figure S6 shows the results of the comparison of porthoDom (domain content similarity cut-off of 0.5, ordering $O1$, domain repeats collapsed) against proteinortho clusters for different numbers of initial k-medoids clusters. The increase in the number of k-medoids clusters is accompanied by an important reduction in computational time. However, the reduction of computational time is also accompanied by a reduced number of identical clusters found between softwares and an increased number of clusters classified as subsets. The raw numbers are available in Table S3.

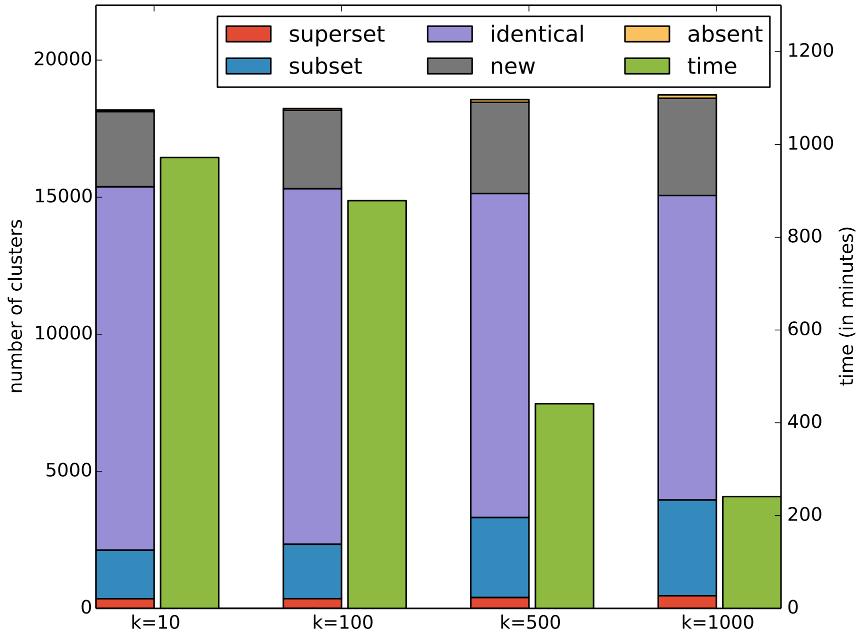

Figure S6: Comparison of porthoDom clustering against proteinortho clustering using different numbers of initial k-medoids. The impact of the number of initial clusters for the creation of the protein domain content similarity search sub-space on proteinortho is represented in this picture. Different values of k-medoids clusters are tested (10, 100, 500, 1000) on a 64-core computer. The numbers of clusters created by porthoDom in each category (superset, subset, identical, new or absent) when compared to proteinortho are reported (left bar of each k-value) as well as the time needed to perform the clustering (right bar of each k-value). Values displayed correspond to mean values of three different runs of porthoDom.

Table S3: Number of clusters in the different evaluation categories for porthoDom when compared against proteinortho with different starting number of k-medoids. Four different initial k-medoids values are tested. For each value, three different runs of porthoDom are performed and the mean values are reported.

classification	number of k-medoids			
	10	100	500	1000
superset	352.7	354	399	462.3
subset	1774	1987.7	2917.7	3498
identical	13255	12970.3	11818.3	11102
new	2742.7	2855.3	3326	3546.7
absent	55.7	63.3	96.7	121.3
